# Patterns of Smoking Behaviour in Low-Income Pregnant Women: A Cohort Study of Differential Effects on Infant Birth Weight

**DOI:** 10.3390/ijerph13111060

**Published:** 2016-10-29

**Authors:** Catherine Hayes, Morgan Kearney, Helen O’Carroll, Lina Zgaga, Michael Geary, Cecily Kelleher

**Affiliations:** 1 Discipline of Public Health and Primary Care, Trinity College Dublin, Dublin 2, Ireland; ZGAGAL@tcd.ie; 2 Department of Obstetrics and Gynaecology, The Rotunda Hospital, Dublin 1, Ireland; morgankearney@gmail.com (M.K.); antenatalmidwife@yahoo.ie (H.O.); mppgeary@gmail.com (M.G.); 3 School of Public Health, Physiotherapy and Sports Science, University College Dublin, Dublin 4, Ireland; cecily.kelleher@ucd.ie

**Keywords:** smoking cessation, inequalities, gender, harm reduction, tobacco control

## Abstract

Maternal smoking accounts for 20%–30% of low birth weight (BW). Second-Hand Smoke (SHS) also negatively affects BW. This cohort study explored the differential effect of smoking patterns during pregnancy on infant BW. Smoking status for 652 self-reported smokers attending public ante-natal clinics was assessed at baseline (V1 first ante-natal visit), 28–32 weeks (V2) and one week after birth (V3). Multivariable generalised linear regression models tested smoking patterns (continuing to smoke, sustained quitting, partial quitting) on BW adjusting for household smoking and other co-variates. Total quitting showed a median increase of 288 g in BW (95% CI (confidence intervals): 153.1–423 g, *p* < 0.001), compared to partial quitting (147 g, (95% CI: 50–244 g), *p* < 0.003). In partial quitters, increased BW was observed only in females 218 g, (95% CI: 81–355 g), *p* = 0.002). Household SHS showed a specific negative influence on pre-term but not term BW. This study suggests that, for low-income women, quitting or partial quitting during pregnancy both have a positive influence on infant BW. Whether others in the household smoke is also important.

## 1. Introduction

The negative relationship between smoking in pregnancy and infant birth weight (BW) is well established. Continued active smoking in pregnancy beyond the first trimester is associated with reduced infant BW [1,2], low birth weight (LBW) and pre-term birth [3], and this relationship is causal and dose dependent [4,5]. The influence of maternal smoking on BW is primarily mediated through fetal growth restriction [5,6] due to utero-placental vasoconstriction caused by nicotine and carbon monoxide poisoning from products of combustion [7]. 

The link between smoking in pregnancy and social disadvantage is also well recognized [8]. Prevalence of smoking in Ireland is highest in young girls of childbearing age with a distinct social class gradient [9], and inequalities in smoking prevalence have increased over time [9,10,11]. These disadvantaged women attend public ante-natal clinics and are at increased risk of having a LBW infant because of high smoking prevalence compounded by disadvantage [12].

From a public health perspective, maternal smoking, because of its particularly high prevalence in socially disadvantaged women, is the leading preventable cause of intra-uterine growth retardation in Europe and North America [5]. Most studies estimate a reduction of 150–300 g in BW in women who continue to smoke compared to non-smoking pregnant women [2,4,13,14,15]. BW declines as tobacco exposure increases; however, the relationship between maternal smoking and BW is not linear; steepest declines in BW have occurred at low levels of exposure measured in the third trimester, suggesting that quitting is far more effective than cutting down [16]. Although not a linear relationship, there is an estimated 27 g reduction in BW for each additional cigarette smoked per day in the third trimester of pregnancy [6].

Even in non-smoking women, high exposure to Second-Hand Smoke (SHS) is negatively associated with BW reduction of 25–75 g and up to 110 g in their offspring [17,18,19,20]. Khazarri et al. [19] found mean BW declined in a dose dependent manner as cotinine levels increased in non-smoking women, and there was no threshold level below which BW was not reduced [19]. 

A number of studies have shown that women who reported quitting smoking early in pregnancy, i.e., prior to four months (16 weeks) gestation, had infants with mean BWs similar to non smokers [13,16,21,22]. The mechanism underpinning this effect is primarily via a reduction in Small for Gestational Age (SGA) [5], and, to a lesser extent, pre-term birth [5,22,23]. Stopping smoking at any time up until 30 weeks has a positive effect on BW, but stopping before 16 weeks has the greatest effect [21].

However, many women quit and relapse repeatedly during pregnancy [24]. An effect of partial, i.e., temporary quitting, may be particularly important for low-income women who are much more likely to continue to smoke during pregnancy [8] and be heavier smokers. This group of smokers is also more likely to be exposed to SHS particularly in their household environment [25,26].

Knowledge of the effect of partial quitting beyond the first trimester on BW is limited. An older study showed that those who stopped smoking temporarily after 16 weeks had some increase in mean BW [21]. An observational study [27] found an estimated gain in BW of 105 g with cutting down by 10 cigarettes per day after the first visit. England et al. concluded, as a result of measuring cotinine levels in the third trimester, that reducing smoking by eight cigarettes per day was necessary to avoid a reduction in BW [28]. The Generation R prospective cohort study of over 7000 pregnant women in the Netherlands showed a small non-significant beneficial effect for reducing the number of cigarettes from ≥5 per day in early pregnancy to <5 per day without quitting [20]. 

Lumley et al. [29] have highlighted the need to obtain greater insight into the experiences of women who continue to smoke so that appropriate interventions and supports may be developed for these women. They raise the possibility of including smoking reduction as a goal in a “harm minimization strategy” similar to other substance misuse strategies. This may have particular importance for low-income women for whom quitting may not be an attainable goal. 

This study aims to determine how variation in patterns of smoking behavior (smoking patterns), defined as: continuing to smoke, partial quitting or sustained quitting during pregnancy in low-income women, who were all smokers at time of pregnancy, influences infant BW, taking into account household smoking, and other smoking and socio-demographic variables including maternal age, parity and infant factors. A secondary objective was to compare smoking patterns with the average number of cigarettes smoked (CiggsAv) at a particular time point as a measure of smoking exposure. Our null hypothesis stated that there was no difference in BW outcome between those who had quit partially and those who continued to smoke during pregnancy, and that smoking pattern and average number of cigarettes smoked were equivalent measures of smoking exposure.

## 2. Materials and Methods

### 2.1. Study Design and Setting

The study is a secondary analysis of data from a large smoking cessation intervention trial which studied the effectiveness of motivational interviewing on smoking cessation in low-income women, delivered by health professionals prenatally and post-partum using a quasi-experimental historical cohort design, described elsewhere [30]. 

### 2.2. Study Population

The study population consisted of a cohort of 1000 expectant mothers attending public ante-natal clinics at the first ante-natal visit in a single large maternity hospital located in Dublin’s north inner city, an area of socio-economic disadvantage, who were smokers at the time of pregnancy and who met the criteria for inclusion in the study (age 16–40, Irish national and resident in North Dublin City and County). Non-national women were excluded to avoid confounding of indigenous differences in smoking prevalence and BWs. A majority of patients belonged to socio-economic groups 5 and 6 [31].

Accurate identification of smokers for participation in the study and assessment of smoking status was based on the work of the Smoke-Free Families project [32]. A smoker at time of pregnancy was defined at the first ante-natal visit as: currently smoking (at least one cigarette in the previous seven days) or smoking at time of pregnancy but had stopped since becoming aware of pregnancy prior to the first ante-natal visit. 

### 2.3. Data Collection and Trial Management

Interviewer administered questionnaires were developed for baseline data collection of demographic and smoking variables at first prenatal visit regardless of gestation (12–18 weeks gestation) (V1) and to record changes in smoking behaviour at the second ante-natal visit (28–32 weeks) (V2), within one week of birth (V3) (used as a proxy for smoking status just prior to delivery) and at two subsequent time points post partum. Only the ante-natal (V1 and V2) and immediate post-partum data (V3) for which complete BW and smoking data were available were used in this analysis, which was carried out retrospectively. 

Demographic details collected at the first ante-natal visit included age, marital status, presence of partner, parity, medical card status, and number of weeks gestation. Smoking history included current smoking status (see above), number of years smoking, amount smoked per day, partner smoking and number of additional smokers in the home. Non-baseline variables were: amount smoked at V2, amount smoked by partner at V2, gestation at birth and single or multiple births. 

### 2.4. Assessment

BW data were extracted from the hospital charts. Self-reporting of smoking abstinence was defined as total abstinence since knowledge of pregnancy (V1) or since previous assessment (V2/V3), in keeping with the “Russell Standard” for reporting outcome criteria in smoking cessation trials [33]. Change in smoking status was based on self-reported response [34]. Self-reported cessation was verified by near patient (point of care) urinary cotinine testing [35] at V2. V2 was chosen as the data point for biochemical validation, and as being the best time point to accurately determine smoking cessation prior to the third trimester.

Smokers were grouped into four categories based on their smoking status at each of the three visits, in keeping with previous research to determine smoking patterns [24]. The categories were as follows:

- Sustained quitters: initial quitters who continued to not smoke across the first three visits (QQQ)
- Continued smokers: initial smokers who continued to smoke across the first three visits (SSS)
- Initial smokers who had quit at V3 (SSQ/SQQ)
- Any other quit attempt during V1 or V2 (QQS/QSQ/QSS/SQS)

Following descriptive analysis, mean BW of “smokers at the first visit but who had subsequently quit” and those who made “any other quit attempt” were combined into a single ‘’partial quitters” category (S or Q) for all subsequent analyses. 

### 2.5. Statistical Analysis

Analyses were carried out using SPSS version 20 (IBM Corp., Armonk, NY, USA) [36]. Descriptive summaries, including means and proportions, were used to describe the characteristics of the study sample, changes in smoking status and number of cigarettes per day. One-way analysis of variance (ANOVA) was carried out to determine statistically significant differences in mean BW according to the derived smoking categories. ANOVA was carried out only on BWs ≥ 1500 g to ensure normality. 

Simple linear regression with BW as the dependent variable was performed on maternal socio-demographic, smoking variables and infant variables as follows: socio-demographic: maternal age, first or subsequent pregnancy, medical card ownership, single parenthood and number of children in the household; smoking: years smoking, current smoking status, amount smoked per day at V1 and V2, partner smoking and amount smoked at V1, number of smokers in the home other than self or partner, and smoking pattern variables; and infant: gestational age at birth, single or multiple birth, and baby’s gender. 

Multiple linear regression modeling was carried out on significant variables as the outcome variable, BW, had a normal distribution after removal of outliers (seven very low BWs < 1500 g in singleton pregnancies). The combined average of the number of cigarettes smoked at V1 and V2 was used. 

As the focus of this analysis was primarily on foetal growth restriction, separate analysis was carried out on BW of term (≥37 weeks) and pre-term (<37 weeks) infants. The unique contribution of each explanatory variable was given by the standardised beta co-efficient and the 95% CI of the estimate.

### 2.6. Ethical Approval

The Rotunda Hospital Research Ethics Committee approved this study. The reference number is 04/01/07.

## 3. Results

**Figure 1:**
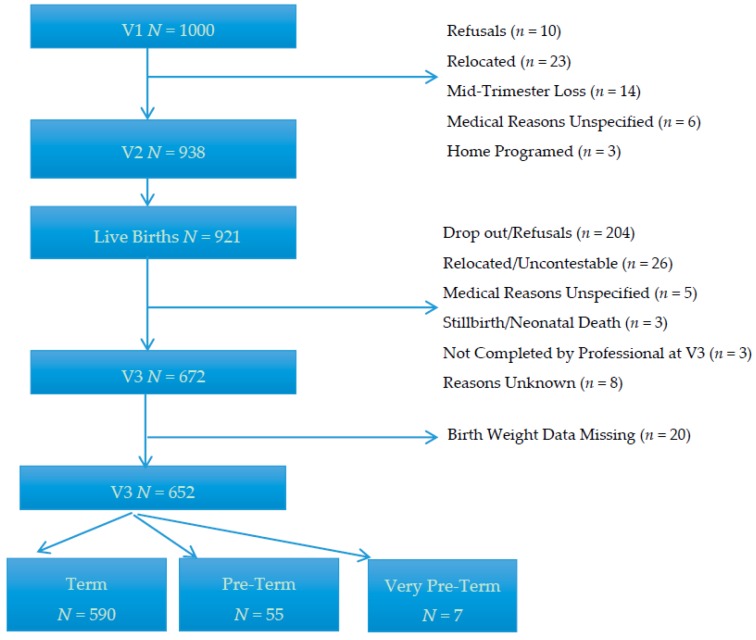
Flow diagram of inclusion and progress of participants.

Figure 1 shows a flow diagram of inclusion and progress of participants throughout the study. Complete data for all three visits was available for 652 women (65.2%). 

### 3.1. Multivariable Analysis

Table 1 shows the prevalence of demographic, smoking and infant variables. No association was shown for maternal age, first or subsequent pregnancy, having a general medical services card, single parenthood, or number of children living in the home. As expected longer gestational age at birth, (direct relationship), female gender and multiple pregnancies (inverse relationship) were significantly associated with BW.

Smoking variables strongly influencing BW were: smoking status at first ante-natal visit (V1) (*p* = 0.001), number of cigarettes smoked at V1 (*p* < 0.001), or V2 (*p* < 0.001), and pattern of smoking across the three visits (*p* < 0.001). Although having a partner who smoked did not affect BW (*p* = 0.3), presence of up to four or more additional smokers in the home was important (*p* = 0.004).

**Figure 2:**
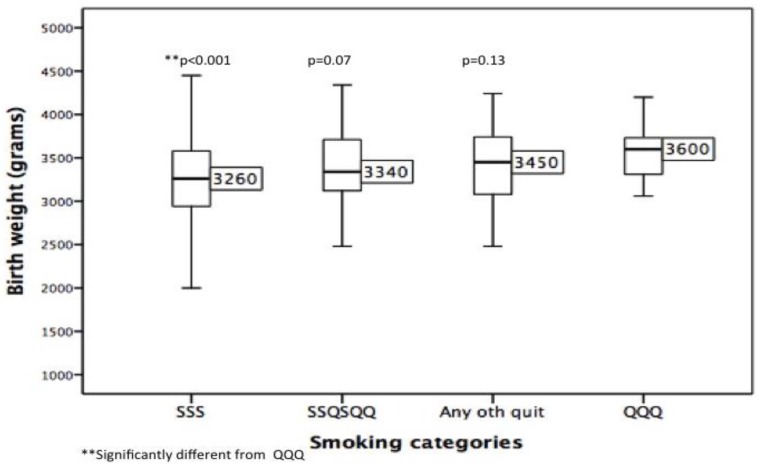
Median birth weight by smoking category (term infants only). Median, interquartile range and minimum and maximum values are shown. SSS = continued smokers; QQQ = sustained quitters; SSQSQQ/Any other quit = partial quitters.

Figure 2 shows median BWs for the four defined smoking patterns for term infants (*n* = 590/652, 90.5%). Overall differences in mean BW between the groups were significant (F = 10.82, degrees of freedom = 2, *p* < 0.001). As expected, the biggest difference in mean BW, (−326 g, (95% CI: −483–(−17) g), *p* < 0.001) was observed between the continued smokers, (SSS, mean BW 3269 g) and those who sustained quitting from the start of pregnancy, (QQQ, mean BW 3595 g), Figure 2. The mean BWs of each category of partial quitters, i.e., “smokers at the first visit who had subsequently quit” (SSQSQQ) and those who made “any other quit attempt” (Any other quit), lay between SSS and QQQ and did not differ significantly from QQQ (Figure 2). The difference between SSS and the partial quitters (SSQSQQ/Any other quit, mean BW 3419 g) was −150 g (95% CI: −261–(−39) g), *p* = 0.008. The sustained quitters were on average 177 g heavier than the partial quitters although this difference did not quite reach statistical significance (95% CI: −5–358 g), *p* = 0.057.

### 3.2. Multivariable Analysis

Table 2 and Table 3 show the contribution of significant factors and co-variates from the univariate analysis on BW for all infants, pre-term and term infants in a multivariable model. Seven cases of extreme LBW (<1500 g) were excluded. Although partner smoking was not significant on univariate analysis, a new variable of “additional home smokers” was created to incorporate partner and additional home smokers (other than mother) to reflect household SHS exposure. 

Two models were developed for all births, term births and preterm births (Table 2 and Table 3). Model 1 tested the effect of smoking pattern on BW, adjusting for gestational age at birth (GA), additional home smokers and gender (Table 2). In Model 2, the weighted average of cigarettes smoked at V1 and V2 (CiggsAv) replaced smoking pattern (Table 3). When the same models were repeated using gestational age instead of BW as the outcome variable, relationships were not significant. 

Model 1 shows that while smoking cessation once pregnancy was known showed the greatest effect of a mean increase of 288 g in BW (95% CI: 153–423 g), *p* < 0.001, partial quitting from the first antenatal visit onwards resulted in approximately half of that increase (147 g, (95% CI: 50–244 g), *p* < 0.003) (Table 2). In Model 2, the combined average number of cigarettes smoked at V1 and V2 had a much smaller inverse effect on BW (−8 g, (95% CI: −11.7–(−5.2) g), *p* < 0.001) (Table 3), compared to smoking pattern throughout pregnancy. The small sample did not show an effect in pre-term infants, although the correct direction of effect was observed for complete and partial quitting in Model 1 and for CiggsAv in Model 2.

### 3.3. Household SHS

Although the separate contribution of household SHS did not demonstrate an overall significant effect on BW, very specific influence in pre-term infants was noted, despite small numbers, in our post hoc analysis of findings. Presence of one or two additional smokers in the home resulted in a smaller mean BW in pre-term infants than homes without additional smokers; Model 1: one additional home smoker (−374 g, (95% CI: −646–(−102) g), *p* = 0.007); two additional smokers (−388 g, (95% CI: −723–(−52) g), *p* = 0.02); Model 2: one additional smoker (−368 g, (95% CI: −643–(−93) g), *p* = 0.009), two additional smokers (−417 g, (95% CI: −747–(−88) g), *p* = 0.01). Numbers were too small to determine effects of SHS from three or more household smokers in pre-term infants even in Model 2, where the number of pre-term infants (35) was substantially higher. In contrast, term babies of mothers with four or more additional smokers in the home were on average 187–195 g smaller than those with no additional home smokers; however, the difference in effect size did not reach statistical significance; (Model 1 (95% CI: −388–14 g), *p* = 0.07); (Model 2 (95% CI: −395–4 g), *p* = 0.05) (Table 2 and Table 3).

### 3.4. Gender

The mean BW for male infants was 3306 g and for females 3166 g—a difference of 140 g. A post hoc gender-stratified analysis was carried out (Table 4) on the variables in Model 1 and Model 2 for term babies only to determine any differential effect of smoking pattern or average number of cigarettes smoked on BW by gender. 

For male and female infants, a significant increase in BW was observed when their mothers had quit throughout pregnancy; males: (347 g (95% CI: 101–593 g), *p* = 0.006); females: (324 g, (95% CI: 149–498 g), *p* < 0.001). A smaller increase in BW was observed in female infants whose mothers partially quit during pregnancy (218 g, (95% CI: 81–355 g), *p* = 0.002), but not in male infants (*p* = 0.3). Differential effects on BW by gender were not observed using CiggsAv as the exposure variable.

Although term babies of mothers from households with four or more additional smokers in the home were lighter than those with no additional home smokers, the effects were not significant: males (Model 1, *p* = 0.095; Model 2: *p* = 0.07), and females (Model 1, *p* = 0.3; Model 2, *p* = 0.4) (Table 4).

The effect of stopping smoking and longer gestation previously observed [5] was not confirmed in our study where no differences in gestational age at birth were noted for infants in either of the three smoking pattern categories, although the lowest minimum value for gestation (21 weeks) was noted in continued smokers. 

## 4. Discussion

Our study examined the combined effect of active and passive smoking throughout pregnancy, complete cessation and partial cessation on BW in a group of low-income women, all of whom were smokers at time of pregnancy. It demonstrated a clear inverse gradient between active smoking pattern and baby’s BW, having adjusted for gestational age, gender, household SHS and other covariates. Sustained quitting resulted in significantly increased BW in term infants of both sexes, with greatest effect in newborn females. An intermediate effect on BW was observed for partial quitters, which impacted favorably on female infant BW. Smoking pattern throughout pregnancy had a greater effect on BW than average number of cigarettes smoked. Post-hoc analysis showed that additional home smoking was selectively inversely associated with a reduction in pre-term though not term infant BW. 

In keeping with previous research, our study provides further evidence that the negative effects of maternal smoking on BW are at least partly reversible [5]. Mothers who continued not to smoke from early in pregnancy experienced greatest benefits in terms of increased BW than those who quit later or not at all. Our findings of a modest increase in BW in partial quitters, compared to continued smokers, further support this concept. The findings add to those of Benjamin–Garner and Stotts [37] in a smaller US study of 225 primarily low-income women, which found a non-significant increase in term infant BW, on cotinine analysis, associated with a reduction from heavy to light smoking exposure during pregnancy.

Our study also showed that smoking pattern throughout pregnancy had a greater effect on BW than average number of cigarettes smoked. Although data completion rates were similar for smoking patterns (88.8%) and CiggsAv (90.5%) in the multivariable models (Table 2 and Table 3), it is likely that data on CiggsAv are less reliable due to underreporting of the amount smoked and regression to the mean. England et al. did not find a significant association between number of cigarettes smoked at enrolment and BW [28]. Their study showed a rapid decline in BW in the third trimester from smoking up to eight cigarettes per day, which then leveled off, implying that greatest damage occurred at lowest levels of exposure.

Our study reports a higher mean difference in BW (−350 g) in term infants of continued smokers and sustained quitters in our low-income cohort than previous general population studies of pregnant women (−250–(−150) g) [3,4,14,15]. The US study in primarily low-income women reported above [37] also found a higher mean difference (−299 g) in BW in term infants of mothers who continued to smoke and those who quit [37], reflecting the combined impact of active and SHS compounded by disadvantage. Although SHS exposure has declined in recent years, the decline has been greatest in socioeconomically advantaged households [26] due to higher smoking rates and a reduced likelihood of having smoke free homes [38]. Benjamin-Garner and Stotts reported 81% of women having partners who continued to smoke or living in households with other smokers [37]. It is possible that quitting is particularly important for low-income pregnant women who suffer multiple environmental stresses including passive smoking related to their socio-economic position. 

In our study, no effect on gestational age at delivery was noted for stopping smoking. This may be due to the exclusion of a small number of very preterm babies from the analysis; however, it is more likely that the study may be underpowered to show an effect on preterm birth.

Current evidence supports a causal relationship between maternal exposure to household environmental tobacco smoke and a small decrease in BW; Ward, adjusted mean difference 36 g, (95% CI: 5–67 g); Leonardi-Bee 33 g (95% CI: 16–51 g) prospective studies; 40 g (95% CI, 26–54 g), retrospective studies; Salmasi −60 g, (95% CI: −80–(−39) g) [14,39,40]. However, it is considered [19] that the 200 g reduction in BW attributable to active smoking may be underestimated by approximately 100 g—the effect of SHS. Few studies have examined the effect of home smoking in addition to partner smoking. In the UK Millennium Cohort study, 10% of households surveyed were shared by non-partner adults e.g., grandparents, who were not captured by that study [14]. The authors of that study acknowledge that the small mean reduction in BW observed of 36 g (95% CI: 5–37 g) from partner smoking is thus an underestimate of the true effect of SHS exposure on BW. It is also possible that partners who smoke may behave differently, i.e., smoke outside in comparison with other household smokers such as grandparents. 

Our study, which examined the cumulative effect of all additional household smokers, did not show an overall effect of domestic SHS on reduced BW. The Generation R study [20] found an effect of passive smoking on BW only in late pregnancy. A selective effect of additional home smoking inversely associated with pre-term infant births (37 weeks) was found although the numbers are small. However, in our study, the influence of household SHS on BW was a post hoc analysis rather than an a priori hypothesis and must be regarded as such in terms of the interpretation of the findings.

However, a cohort study of over 10,000 live singleton births in China, where 49% of men are active smokers, examined the association between passive smoking and pre-term birth. This study showed a 98% increased risk for very pre-term birth <32 weeks (odd ratio (OR) = 1.98, (95% CI: 1.41–2.76 g), *p* for trend = 0.0014). The effect increased with increased duration of exposure but was not shown for moderate pre-term births (32–36 weeks) after adjustment for gestational age [41]. 

A recent meta-analysis of 24 observational studies by Cui et al. [42] reported summary odds ratios (SORs) of preterm birth for women who were sometimes exposed to passive smoking versus women who were never exposed to passive smoking: ever exposed: (SOR = 1.20 g (95% CI = 1.07–1.34 g), I^2^ = 36.1%); never exposed: (SOR = 1.16 g (95% CI: 1.04–1.30 g), I^2^ = 4.4%), respectively. The effect was weaker in the cohort (SOR = 1.10 g, (95% CI: 1.00–1.21 g), *n* = 16) than in cross-sectional studies (SOR = 1.47 g, (95% CI = 1.23–1.74 g), *n* = 5) and higher in Asian populations and studies with more than 100 preterm births. The relationship between passive smoking and pre-term birth needs to be explored in future prospective studies in different populations, and to be examined during different trimesters of pregnancy and by different causes of preterm birth [42]. 

Data on gender-specific associations with BW and smoking during pregnancy and/or Environmental Tobacco Smoke are scarce and somewhat conflicting and compare the effects of the number of cigarettes smoked per day with non-smokers rather than the effects of quitting on BW. 

A German Perinatal Study demonstrated a greater negative effect of maternal smoking on mean BW of newborn girls than boys particularly in heavy smokers (>20 cigaretts per day), [43]. Although a large sample, this study does not distinguish between mothers who smoked for the entire pregnancy and those who stopped early in pregnancy. A disproportionate effect on newborn males has previously been reported in women who smoked more than 10 cigarettes per day (males 8.2% reduction in weight vs. females 4.8%) [44]. More rapid growth of male foetuses and a different hormonal milieu were suggested as the explanation for the greater effect of cigarette smoking on male foetuses. More recent cross-sectional data from 11,000 newborns from the US National Health and Nutrition Survey (NHANES) found ante-natal smoking to be associated with a greater decrease in BW among infant boys who were also more likely to be admitted to intensive care [45].

In our study, the average number of cigarettes smoked did not show a differential effect on male and female BW. However, gender-stratified analysis showed that sustained quitting resulted in significantly increased BW in term infants of both sexes, with the greatest effect in newborn females who are smaller in terms of BW to begin with. Significantly higher BWs were also observed in female infants as a result of partial quitting but not in males. This would suggest a potentially greater impact of quitting on female BW. Gender did not influence BW in pre-term infants. 

The strength of the study lies in its longitudinal design, which facilitated follow-up and measurement of smoking status at a number of time points. Acceptance rates to take part in the study were high, with few refusals (8.1%). Response rate at the second visit in late pregnancy was (93.8%). Urinary cotinine levels validated self-reported smoking cessation at V2. 

Many previous studies have been unable to distinguish between women who have quit before pregnancy and those who have quit since becoming pregnant. Almost all have focused on comparing the effects of smokers with “non-smokers”, i.e., “never smokers” and “ex-smokers” at baseline. All women in our studies were smokers at the time of pregnancy. 

Our study has some limitations worthy of consideration. This was an observational study and has all the hazards of bias and confounding which are often not resolved by multivariable regression analysis. The study did not address certain established confounders such as alcohol intake in pregnancy and the known association with alcohol intake and cigarette smoking. However, Zaren et al. [44] report that although smokers reported a higher alcohol consumption prior to pregnancy, no difference in consumption during pregnancy was found between smokers and non-smokers and overall consumption was low. In addition, we did not have data on maternal body mass index, although very low maternal weight is seldom encountered in Ireland. Unmeasured dietary components such as Vitamin D intake may also have an influence on BW, particularly in low-income women, who may be nutritionally deficient. Iron deficiency anaemia during the first half of pregnancy increases the risk for preterm birth, LBW, infant mortality, and infant iron deficiency [46], and taking iron daily during pregnancy is associated with a significant increase in BW and a reduction in the risk of LBW [47]. Our study did not measure iron intake. 

The second visit took place between 28–32 weeks at the beginning of the third trimester in our study. As the main influence of smoking on BW occurs in the third trimester, our estimate of the average number of cigarettes smoked is likely to be an underestimate of the effect of smoking dose on BW, particularly in heavy smokers. In addition, the timing of partial quitting was not determined. 

In relation to SHS, our study did not consider workplace exposure. However, it took place after the introduction of the ban on smoking in the workplace, hence the household environment is the most likely source of SHS exposure. Similarly, genetic influences were not examined.

## 5. Conclusions

Our large cohort study examined the combined effect of active and passive smoking during pregnancy on BW. It demonstrated a direct relationship around smoking behaviour and BW when smoking behaviour was classified into a number of discrete patterns not entirely explained by the number of cigarettes smoked at particular time points during pregnancy. Smoking pattern had a greater influence on BW than average number of cigarettes smoked. These findings suggest that smoking cessation and at least partial smoking cessation, if total cessation is not possible, is potentially of greater importance for prevention of LBW in low-income women who smoke in pregnancy, due to a myriad of intertwined risk factors related to their socio-economic status. Our preliminary findings suggest a potentially reversible effect of even partial quitting on infant BW, which may be particularly favourable towards improving female BW, and a specific effect of SHS from side-stream smoke on pre-term infant BW, which may be dose related. Harm reduction measures are potentially an important element of tobacco control for this priority group of low-income pregnant smokers and need to be directed towards active and environmental exposure to household smoking. However, research designed to specifically address possible gender-specific influences of total/partial quitting on infant BW and on dose-response effect of household smoking on pre-term BW is needed. 

## Figures and Tables

**Table 1:** Prevalence of demographic and smoking variables (Total *n* = 654).

Factors	*N*	%	Median	Minimum–Maximum	IQR ^a^
**Demographic**	-	-	-	-	-
Maternal Age	654	-	26.0	16–42	9
First Pregnancy	225	34.4	-	-	-
General Medical Services Card	363	55.5	-	-	-
Single Parenthood	236	36.1	-	-	-
Number of Children Living in the Same House	-	-	-	-	-
One Child	227	34.7	-	-	-
Two Children	137	20.9	-	-	-
Three Children	63	9.6	-	-	-
Four or More Children	38	5.8	-	-	-
**Smoking**	-	-	-	-	-
Number of Years Smoking	654	-	10	1–26	9
Smoking Status at First Ante-Natal Visit (V1)	654	-	-	-	-
I smoke now	127	19.5	-	-	-
I smoke now but have cut down since becoming pregnant	449	68.5	-	-	-
I have stopped smoking since I thought I might be pregnant	78	12.0	-	-	-
Number of Cigarettes Smoked/Day at V1	576	-	10	1–60	9
Partner Smoking at V1 (*y*/*n*)	411	62.8	-	-	-
Number of Cigarettes Smoked/Day by Partner at V1	383	-	15	1–60	10
Number Of Cigarettes Smoked/Day at V2	565	-	10	1–60	10
Number Of People Living in House (Other than Self or Partner) Who Smoke	217	-	-	-	-
One Additional Person	121	18.5	-	-	-
Two Additional Persons	58	8.9	-	-	-
Three Additional Persons	18	2.8	-	-	-
Four or More Additional Persons	20	3.1	-	-	-
Smoking Patterns across Three Visits	638	-	-	-	-
SSS ^b^	502	76.8	-	-	-
QQQ ^c^	46	7.0	-	-	-
SSQ/SQQ ^d^	52	8.0	-	-	-
Any other Quit Attempt	40	6.1	-	-	-
**Infant**	-	-	-	-	-
Birth Weight	654	-	3260	800–4800	680
Baby Gender	617	94.3	-		-
Males	306	49.6	-	-	-
Females	311	50.4	-	-	-
Single vs. Multiple	617	94.3	-	-	-
Singleton	610	98.9	-	-	-
Twin	7	1.1	-	-	-
Gestational Age at Birth	635	-	-	26–42	2

**^a^** IQR = interquartile range; ^b^ SSS = continued smokers; ^c^ QQQ = sustained quitters; ^d^ SSQ/SQQ = partial quitters.

**Table 2:** Multivariable analysis of significant variables with birth weight as the dependent variable—MODEL1—Smoking pattern throughout pregnancy ^a^.

Variable		All				Pre Term < 37 Weeks				Term ≥ 37 Weeks			
		*N*	*Beta*	95% CI ^b^	*p* *	*N*	*Beta*	95% CI ^b^	*p* *	*N*	*Beta*	95% CI ^b,^*	*p* *
Smoking Categories across Three Visits		579	-	-	-	35	-	-	-	544	-	-	-
QQQ		42	288.0	153.1–423.0	<0.001 *	5	67.2	−272.8–407.2	0.70	37	327.4	183.2–471.7	<0.001 *
S/Q		88	146.9	49.5–244.1	0.003 *	3	181.6	−236.5–599.8	0.40	85	146.2	46.5–245.9	0.004 *
SSS (reference)		449	-	-	-	27	-	-	-	422	-	-	-
Additional Home Smokers	Four or More Home Smokers	22	−170.5	−361.4–20.5	0.08	2	−43.6	−547.0–459.8	0.87	20	−186.8	−387.8–14.2	0.07
	Three Home Smokers	40	58.9	−89.7–207.4	0.44	2	−280.4	−806.6–245.7	0.30	38	82.3	−71.4–236.0	0.29
	Two Home Smokers	81	−93.4	−208.5–21.7	0.11	7	−387.6	−723.1–(−52.1)	0.02 *	74	−74.2	−194.8–46.5	0.23
	One Home Smoker	288	−25.0	−109.3–59.2	0.56	13	−374.2	−646.4–(102.0)	0.007 *	275	−7.2	−94.7–80.3	0.87
	No Home Smokers (reference)	148	-	-	-	11	-	-	-	137	-	-	-
Baby Gender	Females	294	−155.1	−224.6–(−85.7)	<0.001 *	18	−59.6	−293.4–174.2	0.62	276	−159.0	−231.0–(−87.0)	<0.001 *
	Males (reference)	285	-	-	-	17	-	-	-	268	-	-	-
Gestational Age at Birth		579	161.2	-	<0.001 *	35	124.5	56.9–192.2	<0.001 *	544	149.4	120.9–177.8	<0.001 *

^a^ Excluding 7 cases of birth weight < 1500 g; * *p* < 0.05; CI ^b^ = Confidence Intervals.

**Table 3:** Multivariable analysis of significant variables with birth weight as the dependent variable—MODEL2—Average number of cigarettes smoked at V1 and V2 ^a^.

Variable		All				Pre Term < 37 Weeks				Term ≥ 37 Weeks			
		*N*	*Beta*	95% CI ^b^	*p* *	*N*	*Beta*	95% CI ^b^	*p* *	*N*	*Beta*	95% CI ^b^^,^*	*p* *
Average number cigarettes Smoked at V1 and V2 (CiggsAv)		590	−8.4	−11.7–(−5.2)	<0.001 *	35	−1.7	−12.0–8.5	0.74	555	−8.9	−12.4–(−5.5)	<0.001 *
Additional Home Smokers	Four or More Home Smokers	22	−180.3	−369.5–8.8	0.06	2	−73.1	−575.6–429.3	0.78	20	−195.4	−394.5–3.7	0.05
	Three Home Smokers	41	55.8	−90.3–201.8	0.45	2	−301.3	−825.6–223.0	0.26	39	77.5	−73.4–228.4	0.31
	Two Home Smokers	84	−102.1	−214.9–10.7	0.08	7	−417.2	−747.0–(−87.5)	0.01 *	77	−80.7	−198.8–37.5	0.18
	One Home Smoker	291	−24.7	−107.7–58.3	0.56	13	−368.3	−643.1–(−93.4)	0.009 *	278	−7.3	−93.5–78.8	0.87
	No Home Smokers (reference)	152	-	-	-	11	-	-	-	141	-	-	-
Baby Gender	Females	296	−141.5	−09.9–(−73.2)	<0.001 *	18	−59.7	−295.9–176.4	0.62	278	−142.5	−213.4–(−71.7)	<0.001 *
	Males (reference)	294	-	-	-	17	-	-	-	277	-	-	-
Gestational Age at Birth		590	159.9	141.1–178.8	<0.001 *	35	119.9	54.3–185.4	<0.001 *	555	150.0	121.7–178.2	<0.001 *

^a^ Excluding 7 cases of birth weight < 1500 g; * *p* < 0.05; CI ^b^ = Confidence Intervals.

**Table 4:** Multivariable analysis of smoking pattern and average number of cigarettes smoked—MODELS 1 and 2 by gender (term babies only).

Model		Males				Females			
		*N*	*Beta*	95% CI ^a^	*p* *	*N*	*Beta*	95% CI ^a^	*p* *
MODEL 1		-	-	-	-	-	-	-	-
Smoking Categories across Three Visits	QQQ	13	346.7	100.6–592.7	0.006 *	24	323.5	148.6–498.4	<0.001 *
	S/Q	42	66.7	−78.1–211.6	0.37	43	218.0	80.9–355.1	0.002
	SSS (reference)	213	-	-	-	209	-	-	-
Additional Home Smokers	Four or More Home Smokers	10	−248.0	−539.2–43.2	0.095	10	−137.3	−413.0–138.4	0.33
	Three Home Smokers	22	58.3	−153.7–270.4	0.59	16	119.7	−105.3–344.7	0.30
	Two home smokers	36	−26.8	−205.0–151.5	0.77	38	−104.6	−269.0–59.8	0.21
	One home smoker	134	−26.9	−156.7–102.9	0.69	141	15.4	−102.8–133.6	0.80
	No Home Smokers (reference)	66	-	-	-	71	-	-	-
Gestational Age at Birth		268	154.1	113.4–194.8	<0.001 *	276	143.3	103.0–183.5	<0.001 *
MODEL 2		-	-	-	-	-	-	-	-
Average number Cigarettes Smoked at V1 and V2		277	−9.5	−14.3–(−4.7)	0.000	278	−8.1	−13.0–(−3.2)	0.001
Additional Home Smokers	Four or More Home Smokers	10	−261.9	−547.2–23.4	0.072	10	−128.8	−405.9–148.2	0.36
	Three Home Smokers	23	65.3	−138.6–269.1	0.53	16	96.5	−131.1–324.2	0.41
	Two Home Smokers	39	−45.2	−215.8–125.4	0.60	38	−118.3	−284.4–47.7	0.16
	One Home Smoker	136	−18.8	−144.2–106.7	0.77	142	4.7	−114.3–123.8	0.94
	No Home Smokers (reference)	69	-	-	-	72	-	-	-
Gestational Age at Birth		277	153.5	113.9–193.2	<0.001 *	278	146.9	106.2–187.6	<0.001 *

* *p* < 0.05; ^a^ CI = Confidence Intervals.

## References

[1] Butler C.C., Rollnick S., Cohen D., Bachmann M., Russell I., Stott N. (1999). Motivational consulting versus brief advice for smokers in general practice: A randomized trial. Br. J. Gen. Pract..

[2] Lindley A.A., Becker S., Gray R.H., Herman A.A. (2000). Effect of continuing or stopping smoking during pregnancy on infant birth weight, crown-heel length, head circumference, ponderal index, and brain: Body weight ratio. Am. J. Epidemiol..

[3] Jaddoe V.W., Verburg B.O., De Ridder M., Hofman A., Mackenbach J.P., Moll H.A., Steegers E.A., Witteman J.C. (2007). Maternal smoking and fetal growth characteristics in different periods of pregnancy. Am. J. Epidemiol..

[4] U.S. Department of Health and Human Services (2004). The Health Consequences of Smoking. A Report of the Surgeon General. Chapter 5: Reproductive Effects.

[5] Cnattingius S. (2004). The epidemiology of smoking during pregnancy: Smoking prevalence, maternal characteristics, and pregnancy outcomes. Nicotine Tob. Res..

[6] Salihu H.M., Wilson R.E. (2007). Epidemiology of prenatal smoking and perinatal outcomes. Early Hum. Dev..

[7] Andersen M.R., Simonsen U., Uldbjerg N., Aalkjaer C., Stender S. (2009). Smoking cessation early in pregnancy and birth weight, length, head circumference, and endothelial nitric oxide synthase activity in umbilical and chorionic vessels: An observational study of healthy singleton pregnancies. Circulation.

[8] Hiscock R., Bauld L., Amos A., Fidler J.A., Munafò M. (2012). Socioeconomic status and smoking: A review. Ann. N. Y. Acad. Sci..

[9] Morgan K., McGee H., Watson D., Perry I., Barry M., Shelley E., Harrington J., Molcho M., Layte R., Tully N. (2008). SLAN 2007: Survey of Lifestyle, Attitudes & Nutrition in Ireland: Main Report.

[10] Friel S., Nic Gabhainn S., Kelleher C. (1999). The National Health and Lifestyle Surveys: Survey of Lifestyle, Attitudes and Nutrition (SLAN) & the Irish Health Behaviour in School-Aged Children Survey (HBSC).

[11] Kelleher C., Friel S., Nic Gabhainn S., Corrigan H., Nolan G., Sixsmith J., Walsh O., Cooke M. (2003). The National Health and Lifestyle Surveys: Survey of Lifestyle, Attitudes and Nutrition (SLAN) & the Irish Health Behaviour in School-Aged Children Survey (HBSC).

[12] Murrin C., Segonds-Pichon A., Fallon U.B., Hannon F., Bury G., Loftus B.G., Murphy A.W., Morrison J.J., Daly S., Kelleher C.C. (2007). Self-reported pre-pregnancy maternal body mass index and infant birth-weight. Ir. Med. J..

[13] Butler N.R., Goldstein H., Ross E.M. (1972). Cigarette smoking in pregnancy: Its influence on birth weight and perinatal mortality. Br. Med. J..

[14] Ward C., Lewis S., Coleman T. (2007). Prevalence of maternal smoking and environmental tobacco smoke exposure during pregnancy and impact on birth weight: Retrospective study using millennium cohort. BMC Public Health.

[15] U.S. Department of Health and Human Services (2014). The Health Consequences of Smoking—50 Years of Progress. A Report of the Surgeon General.

[16] England L.J., Kendrick J.S., Gargiullo P.M., Zahniser S.C., Hannon W.H. (2001). Measures of maternal tobacco exposure and infant birth weight at term. Am. J. Epidemiol..

[17] Windham G.C., Hopkins B., Fenster L., Swan S.H. (2000). Prenatal active or passive tobacco smoke exposure and the risk of preterm delivery or low birth weight. Epidemiology.

[18] Misra D.P., Nguyen R.H.N. (1999). Environmental tobacco smoke and low birth weight: A hazard in the workplace?. Environ Health Perspect. Suppl..

[19] Kharrazi M., DeLorenze G.N., Kaufman F.L., Eskenazi B., Bernert J.T., Graham S., Pearl M., Pirkle J. (2004). Environmental tobacco smoke and pregnancy outcome. Epidemiology.

[20] Jaddoe V.W.V., Troe E.-J.W.M., Hofman A., Mackenbach J.P., Moll H.A., Steegers E.A.P., Witteman J.C.M. (2008). Active and passive maternal smoking during pregnancy and the risks of low birthweight and preterm birth: The generation R study. Paediatr. Perinat. Epidemiol..

[21] MacArthur C., Knox E.G. (1988). Smoking in pregnancy: Effects of stopping at different stages. Br. J. Obstet. Gynaecol..

[22] McCowan L.M., Dekker G.A., Chan E., Stewart A., Chappell L.C., Hunter M., Moss-Morris R., North R.A. (2009). Spontaneous preterm birth and small for gestational age infants in women who stop smoking early in pregnancy: Prospective cohort study. Br. Med. J..

[23] Li C.Q., Windsor R.A., Perkins L., Goldenberg R.L., Lowe J.B. (1993). The impact on infant birth weight and gestational age of cotinine-validated smoking reduction during pregnancy. JAMA.

[24] Pickett K.E., Wakschlag L.S., Dai L., Leventhal B.L. (2003). Fluctuations of maternal smoking during pregnancy. Obstet. Gynecol..

[25] Rowa-Dewar N., Lumsdaine C., Amos A. (2015). Protecting children from smoke exposure in disadvantaged homes. Nicotine Tob. Res..

[26] Moore G.F., Currie D., Gilmore G., Holliday J.C., Moore L. (2012). Socioeconomic inequalities in childhood exposure to secondhand smoke before and after smoke-free legislation in three UK countries. J. Public Health.

[27] Secker-Walker R.H., Vacek P.M., Flynn B.S., Mead P.B. (1998). Estimated gains in birth weight associated with reductions in smoking during pregnancy. J. Reprod. Med..

[28] England L.J., Kendrick J.S., Wilson H.G., Merritt R.K., Gargiullo P.M., Zahniser S.C. (2001). Effects of smoking reduction during pregnancy on the birth weight of term infants. Am. J. Epidemiol..

[29] Lumley J., Chamberlain C., Dowswell T., Oliver S., Oakley L., Watson L. Interventions for Promoting Smoking Cessation during Pregnancy. http://onlinelibrary.wiley.com/doi/10.1002/14651858.CD001055.pub4/abstract.

[30] Hayes C.B., Collins C., O’Carroll H., Wyse E., Gunning M., Geary M., Kelleher C.C. (2013). Effectiveness of motivational interviewing in influencing smoking cessation in pregnant and postpartum disadvantaged women. Nicotine Tob. Res..

[31] Geary M. (2004). Clinical Report.

[32] Melvin C.L., Tucker P. (2000). Measurement and definition for smoking cessation intervention research: The smoke-free families experience. Tob. Control.

[33] West R., Hajek P., Stead L., Stapleton J. (2005). Outcome criteria in smoking cessation trials: Proposal for a common standard. Addiction.

[34] Abrahamsson A., Ejlertsson G. (2000). Smoking patterns during pregnancy: Differences in socioeconomic and health-related variables. Eur. J. Public Health.

[35] Cope G., Nayyar P., Holder R., Gibbons J., Bunce R. (1996). A simple near-patient test for nicotine and its metabolites in urine to assess smoking habit. Clin. Chim. Acta.

[36] IBM Corp. (2011). IBM SPSS Statistics for Windows, Version 20.

[37] Benjamin-Garner R., Stotts A. (2013). Impact of smoking exposure change on infant birth weight among a cohort of women in a prenatal smoking cessation study. Nicotine Tob. Res..

[38] Phillips R., Amos A., Ritchie D., Cunningham-Burley S., Martin C. (2007). Smoking in the home after the smoke-free legislation in scotland: Qualitative study. Br. Med. J..

[39] Leonardi-Bee J., Smyth A., Britton J., Coleman T. (2008). Environmental tobacco smoke and fetal health: Systematic review and meta-analysis. Arch. Dis. Child. Fetal Neonatal Ed..

[40] Salmasi G., Grady R., Jones J., McDonald S.D. (2010). Environmental tobacco smoke exposure and perinatal outcomes: A systematic review and meta-analyses. Acta Obstet. Gynecol. Scand..

[41] Qiu J., He X.C., Cui H.M., Zhang C., Zhang H.H., Dang Y., Han X.D., Chen Y., Tang Z.F., Zhang H.R. (2014). Passive smoking and preterm birth in urban china editorial comment. Obstet. Gynecol. Surv..

[42] Cui H., Gong T.T., Liu C.X., Wu Q.J. (2016). Associations between passive maternal smoking during pregnancy and preterm birth: Evidence from a meta-analysis of observational studies. PLoS ONE.

[43] Voigt M., Hermanussen M., Wittwer-Backofen U., Fusch C., Hesse V. (2006). Sex-specific differences in birth weight due to maternal smoking during pregnancy. Eur. J. Pediatr..

[44] Zarén B., Lindmark G., Bakketeig L. (2000). Maternal smoking affects fetal growth more in the male fetus. Paediatr. Perinat. Epidemiol..

[45] Tayie F.A., Powell C. (2012). Sex differences in the association between prenatal smoking and decreased birthweight, and intensive health care of the neonate. Behav. Med..

[46] Zimmermann M.B., Hurrell R.F. (2007). Nutritional iron deficiency. Lancet.

[47] Krafft A. (2013). Iron supplementation in pregnancy. Br. Med. J..

